# Practical Departments

**Published:** 1902-02-01

**Authors:** 


### PRACTICAL DEPARTMENTS.

### NEW CONSULTING-ROOM COUCH.

In response to the suggestion of a gentleman in the medical profession, Messrs. E. Shaw & Co., of 13 Piccadilly, Manchester, have designed and built a new consulting-room couch. The idea being that most of the couches in use stand too high, so that the patient has to be lifted up and down, this new contrivance has a system of screws, so that the patient may first take the desired position, after which the couch may be raised to the necessary height, and lowered again when the examination is over. In its resting position the couch is 2 feet from the ground, and in arranging the elevation mechanism the varying height of medical men has been taken into consideration. The cushion is of American leather, and the head is adjustable. Two sets of legs are supplied with each couch, one with castors and one without.

### BOVRIL.

A reception to which members of the medical profession were invited, took place on Thursday, 23rd inst., at the factories of Bovril, Limited, when the visitors were conducted over the buildings, and the processes of manufacture demonstrated. The beef for the production of Bovril comes from Canada in hermetically sealed tins, and throughout the process of making, machinery is employed, the food not being touched by hand at all. The nutritive virtue of Bovril lies in the addition of albumin and fibrine to the beef extract, and for this fresh beef is used, rather than the exhausted meat from which the juices have been already extracted. The utmost care is taken to ensure cleanliness in all the processes; and the machinery used is of the most modern. Among other products shown on Thursday were some Government rations and Arctic expedition preserved foods.

